# Concurrent Longitudinal Extensive Transverse Myelitis and Leptomeningitis in West Nile Virus: A Report of a Rare Case

**DOI:** 10.7759/cureus.53705

**Published:** 2024-02-06

**Authors:** Ivanna Joseph, Diamler Vadlamuri, Ivia E Rivera Agosto, Mehdi Ghasemi

**Affiliations:** 1 Department of Neurology, Lahey Hospital and Medical Center, Burlington, USA

**Keywords:** lower limb paralysis, intravenous immunoglobulin (ivig), leptomeningitis, longitudinal extensive transverse myelitis, neuroinvasive west nile virus

## Abstract

Here we report a rare case with concurrent longitudinal extensive transverse myelitis (LETM) and leptomeningitis due to West Nile virus infection. A 47-year-old man initially presented with a six-day progressive, intermittent low-grade fever, headache, diplopia, malaise, myalgia, lower back pain, and difficulty walking that developed into progressive asymmetric paralysis. Initial lab work was notable for mild lactic acidosis and hyperCKemia. Brain MRI with contrast demonstrated small foci of leptomeningeal enhancement in the cerebellum, pons, medulla, and right CN VI at the cisternal segment. MRI of the spine was remarkable for edema in the spinal cord extending from T_10_ to L_1_ with diffuse enlargement of the cord contour at T_11_ to L_1_ and subtle enhancement of nerve roots within the thecal sac and cauda equina regions. The patient responded partially to five-day intravenous immunoglobulin therapy (total dose, 2 g/kg). Electromyography four months after the onset of symptoms also showed chronic reinnervation with active denervating features in thoracolumbar myotomes. Clinically, this case highlights the ill-defined and non-specific nature of the presentation of West Nile neuroinvasive disease. It can pose a diagnostic challenge for clinicians and, if unrecognized, is associated with significant morbidity and mortality in older and compromised individuals.

## Introduction

West Nile virus (WNV) is a mosquito-borne flavivirus and human, equine, and avian neuropathogen [1]. Birds serve as the predominant hosts and WNV is maintained in nature primarily involving the Culex species mosquitoes in a mosquito-bird-mosquito transmission cycle. Humans are also known hosts of the virus with the first recognized outbreak of WNV occurring in Israel in 1951 with a total of 123 reported cases [2]. Today, according to the Centers for Disease Control and Prevention (CDC), WNV is the most common mosquito-borne disease in the United States, with an average of 2,205 cases reported each year [3].

Most people who are infected with WNV do not have any symptoms. About 20% of them develop an acute systemic febrile illness, and less than 1% develop severe neurological complications, such as meningitis or encephalitis [4]. Of the neuroinvasive diseases, it is important to recognize that a minority of patients can also present in atypical fashion with acute onset weakness or flaccid paralysis as a result of longitudinal extensive transverse myelitis (LETM) [5-7]. LETM is defined as lesions visualized on a spinal MRI extending over three or more adjoining vertebral segments [8]. It is important to consider this in the differential diagnosis of acute flaccid paralysis as serological confirmation of WNV can aid in early symptomatic management and interventions aimed at halting the progression of the disease. In this case report, we describe a rare case of a patient who had concurrent LETM and leptomeningitis as a result of WNV infection.

## Case presentation

A 47-year-old man with no previous significant medical history or recent travel presented to the emergency department (ED) in late August 2023 with a six-day progressive, intermittent low-grade fever, headache, malaise, myalgia, double vision, lower back pain, and difficulty walking due to the lower extremity weakness. He reported that his lower back pain began six days ago, while he was playing tennis. He felt "a taking a hard step and feeling a twinge in his back". He continued to play tennis the following day, but he experienced some strain on his lower back and had difficulty bending down to pick up balls due to the pain. The following day, he felt muscle aches in his lower legs, especially in his calves. On the third day before ED admission, he had difficulty climbing stairs and his lower extremity weakness worsened on the fourth day. One day before admission, he also developed horizontal double vision as well as a constant, pressure-like headache on the vertex, with a severity of 7/10 and no relief from ibuprofen. The patient did not have bladder or bowel movement issues.

At the initial presentation, he had a temperature of 102.8 ^o^F. He had an unremarkable heart, chest, and abdomen exam. On neurologic examination, speech was fluent, cranial nerves (CN) 2 through 12 demonstrated horizontal diplopia on looking to his right more than the left side and ophthalmoplegia on lateral gaze (suggestive of CN 6 palsy). Bilateral upper extremity and left lower extremity strength were 5 out of 5. Right lower extremity strength was 3 out of 5 for hip flexion, knee flexion, and knee extension, whereas it was 4 out of 5 for ankle dorsi flexion and plantar flexion. The sensation was grossly intact. Deep tendon reflexes were 1+ on bilateral biceps, triceps, and brachioradialis, and left Achilles and patella; however, right Achilles and patellar reflexes were absent. Babinski’s sign was present bilaterally. The patient was unable to stand without assistance and had trouble walking. Finger-nose-finger showed no signs of dysmetria or ataxia.

As shown in Table 1, initial laboratory testing was only remarkable for mild lactic acidosis, elevated C-reactive protein, and creatine kinase. Workup was negative for blood culture (2X), Lyme, HIV, HTLV I/II, hepatitis, arbovirus, tuberculosis, syphilis, and Herpes simplex viruses as well as antinuclear antibody (ANA) screen, and anti-myelin oligodendrocyte glycoprotein (MOG), -aquaporin-4 (AQP4 or NMO-IgG), and -Sjogren (Ro/La) antibodies.

**Table 1:** The results of initial blood testing in the presented case with West Nile virus neuroinfection. ALT, alanine transaminase; AST, aspartate aminotransferase; BUN, blood urea nitrogen; CK, creatine kinase; HIV, human insufficiency virus; HTLV, human T-lymphotropic virus; MOG, myelin oligodendrocyte glycoprotein.

Measurements	Results	Reference Range
WBC Count	10.83	4.00 - 11.00 K/uL
RBC Count	4.99	4.10 - 5.60 M/uL
Platelet Count	235	150 - 450 K/uL
Sodium	135	134 - 144 mmol/L
Potassium	4	3.2 - 5.1 mmol/L
Chloride	100	97 - 109 mmol/L
BUN	16	9 - 26 mg/dL
Creatinine	1.3	0.7 - 1.3 mg/dL
Glucose	126	70 - 110 mg/dL
Calcium	9.1	8.5 - 10.5 mg/dL
Magnesium	1.9	1.8 - 2.4 mg/dL
AST	37	15 - 37 IU/L
ALT	25	0 - 55 IU/L
CK	868	30 - 200 IU/L
C-Reactive Protein	6.1	< 5.0 mg/L
TSH	0.63	0.35 - 4.94 uIU/mL
Angiotensin Converting Enzyme	13.2	9-67 units/L
Vitamin B12	284	213 - 816 pg/mL
HbA1C	5.1	4.6 - 5.6 %
Lactic Acid	2.6	0.5 - 2.0 mmol/L
Antinuclear Antibody screen	Negative	Negative
Anti-SSA(RO) Antibody	1	<1.0 AI
Anti-SSA(LA) Antibody	<1.0	<1.0 AI
Anti-MOG Antibody	Negative	Negative
Anti-aquaporin-4 (AQP4) Antibody	Negative	Negative
West Nile IgM Antibody	Positive	Negative
West Nile IgG Antibody	Negative	Negative
Eastern equine encephalitis IgM Antibody	Negative	Negative
Hepatitis Antibody Panel	Non-Reactive	Non-Reactive
Blood Culture 2X	No growth after five days	No growth after five days
Lyme Antibody Screen	Negative	Negative
HIV 1/2 Antibody	Negative	Negative
Treponema Pallidum IgG & IgM Antibodies	Non-Reactive	Non-reactive
HTLV I/II Antibody	Negative	Negative
Herpes Simplex Virus PCR	Negative	Negative
Quantiferon TB Gold Plus	Negative	Negative

**Figure 1:**
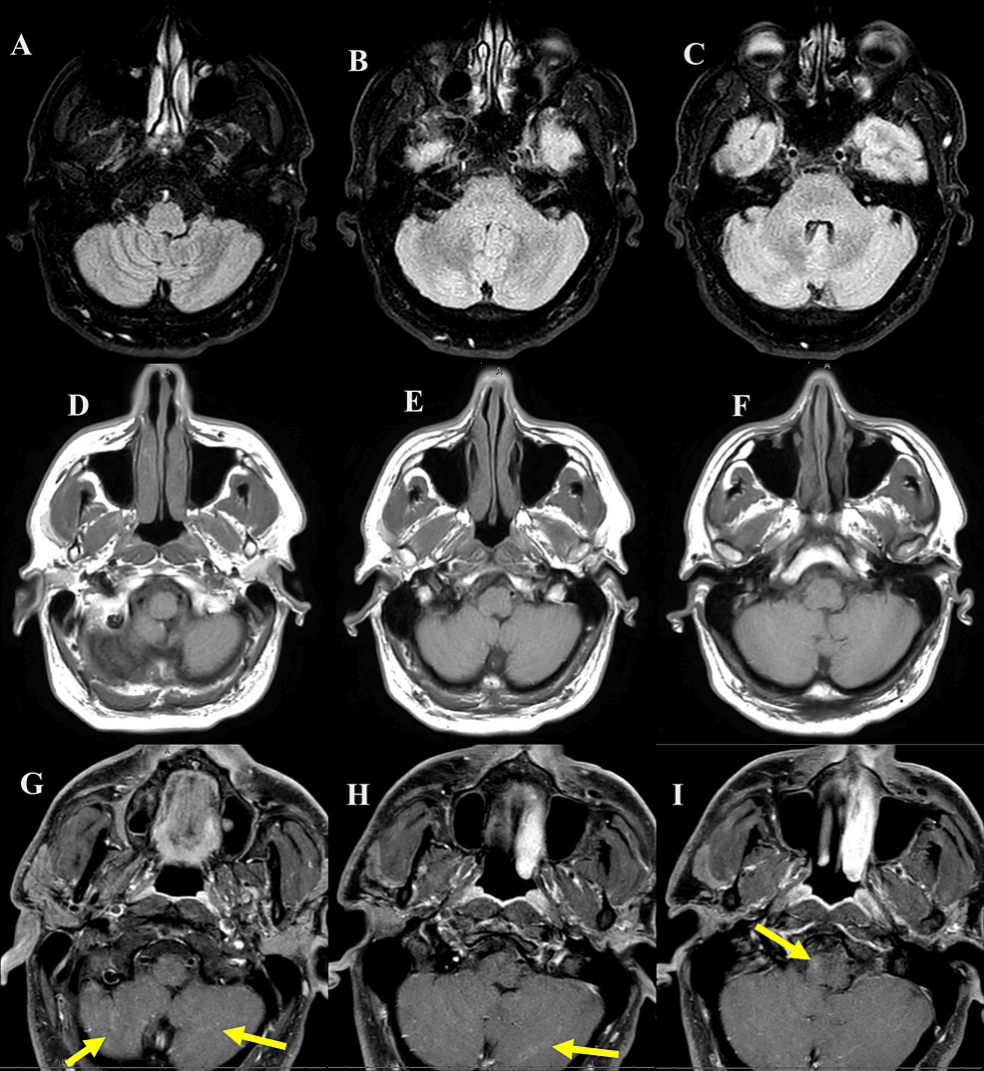
Axial FLAIR (A-C), axial T1 pre- (D-F), and post-contrast (G-I) brain magnetic resonance imaging (MRI) from day 2 of hospitalization. The imaging shows multiple tiny linear and punctate foci of subtle enhancement in the cerebellum especially inferiorly (possibly representing a small foci of leptomeningeal enhancement) and subtle punctate enhancement in the pons and medulla (yellow arrows). FLAIR: Fluid-attenuated inversion recovery

The brain MRI with contrast (day 2 hospitalization) demonstrated multiple tiny linear and punctate foci of subtle enhancement in the cerebellum especially inferiorly (possibly representing a small foci of leptomeningeal enhancement), subtle punctate enhancement in the pons and medulla, and minimal enhancement of the right CN VI at the cisternal segment (Figure 1).

**Figure 2:**
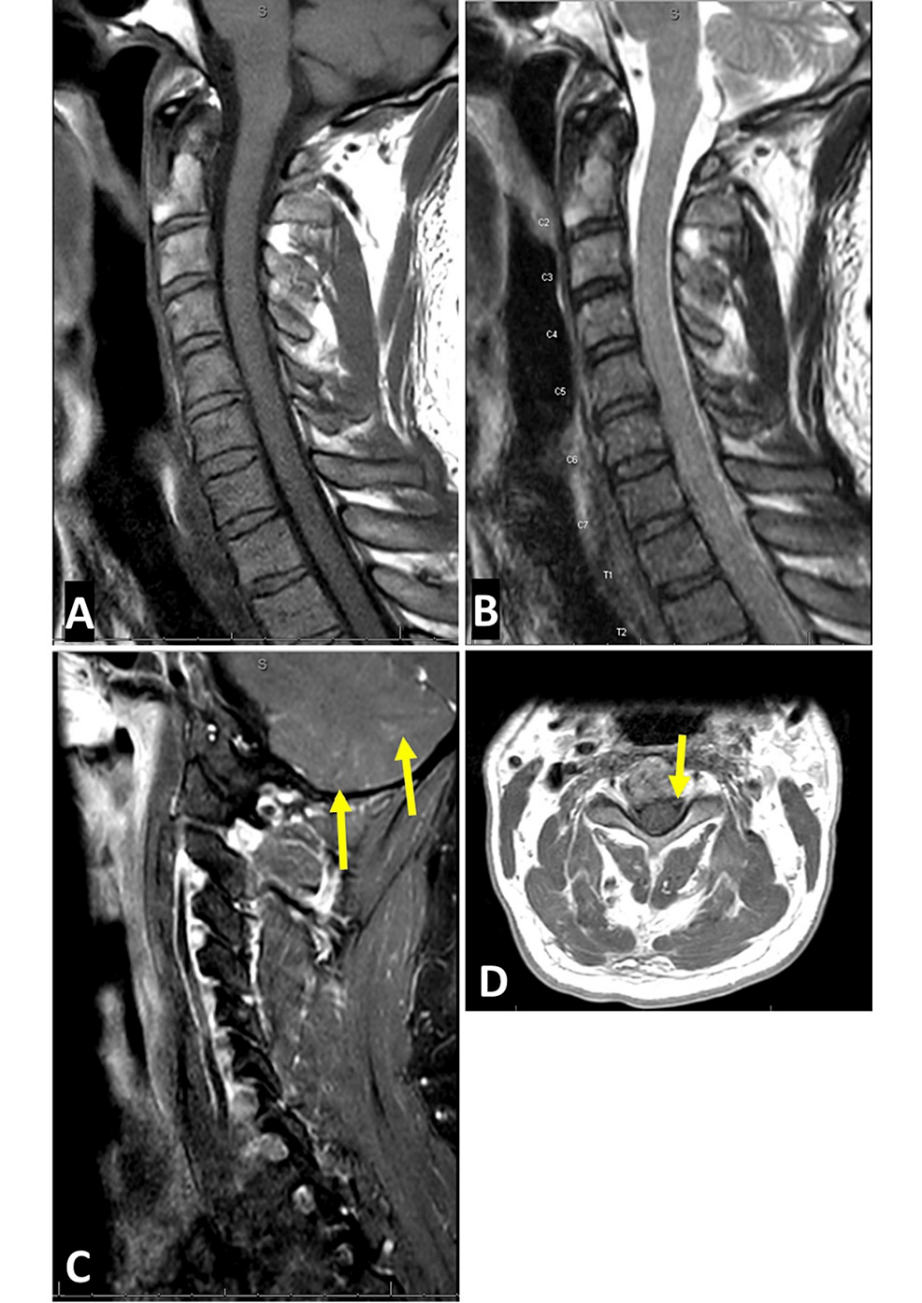
Sagittal T2 (A), T1 pre- (B) and post-contrast (C), as well as axial T1 post-contrast (D) MRI cervical spine from day 2 of hospitalization. The imaging shows subtle leptomeningeal enhancement surrounding the spinal cord and possibly some of the nerve roots. There is also linear and possibly nodular enhancement within the cerebellum predominantly in the cerebellar sulci suggestive of leptomeningeal enhancement (yellow arrow).

**Figure 3:**
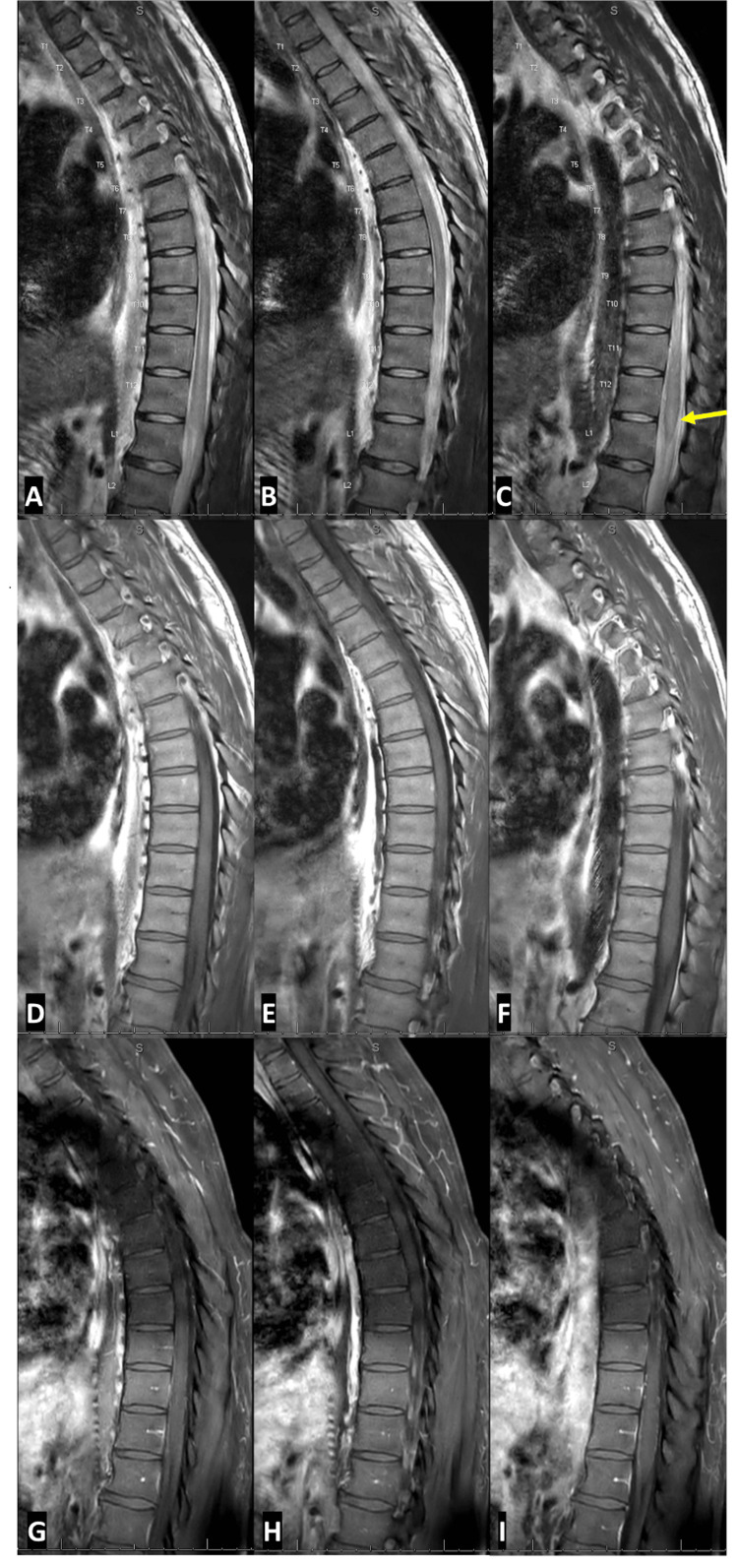
Sagittal T2 (A-C), T1 pre- (D-F), and post-contrast (G-I) MRI thoracic spine from day 2 of hospitalization. The imaging shows edema in the spinal cord extending from T10 to L1 with diffuse enlargement of the cord contour at T11 to L1 (yellow arrow) and without enhancement, suggestive of longitudinally extensive transverse myelitis or LETM.

Cervical/thoracic/lumbosacral spine MRI with contrast (Figures 2, 3) also demonstrated edema in the spinal cord extending from T10 to L1 with diffuse enlargement of the cord contour at T11 to L1 (suggestive of longitudinally extensive transverse myelitis or LETM), and subtle enhancement of nerve roots within the thecal sac and cauda equina regions.

As shown in Table 2, the cerebrospinal fluid (CSF) study (day 2 of hospitalization) revealed elevated WBC of 247 cells/µL (with lymphocyte predominance, 63%) and protein level (86 mg/dL), with normal glucose. Infectious workup confirmed infection with WNL with positive CSF and serum IgM and negative serum IgG.

**Table 2:** The results of cerebrospinal fluid (CSF) study on day 2 of hospitalization in the presented case with West Nile virus neuroinfection. MOG, myelin oligodendrocyte glycoprotein; VDRL, venereal disease research laboratory. * Meningitis/Encephalitis Panel by PCR includes Cytomegalovirus, Cryptococcus neoformans/gattii, Escherichia coli K1, Enterovirus, Haemophilus influenzae, Human herpesvirus 6, Herpes simplex virus 1, Herpes simplex virus 2, Listeria monocytogenes, Neisseria meningitidis, Human parechovirus, Streptococcus agalactiae, Varicella zoster virus, and Streptococcus pneumoniae. ** Encephalitis/Paraneoplastic Autoantibody Panel includes ANNA1 (HU), ANNA2 (RI), ANNA3, PCA1 (YO), PCA2, PCA TR (DNER), AGNA/SOX1 Glial nuclear type 1, Amphiphysin, CRMP5/CV2, GAD65, MA2/TA, AQP4, and Myelin antibodies.

Measurements	Results	Reference Range
WBC Count	247	≤10 cells/uL
Differentials	63% Lymphocytes	<65%
RBC Count	<500	<2000 cells/uL
Protein	86	15 - 45 mg/dL
Glucose	55	40 - 70 mg/dL
Myelin Basic Protein	28.5	0.00 - 5.50 ng/mL
VDRL	Non-Reactive	Non-Reactive
Angiotensin Converting Enzyme	3	≤15 U/L
Anti-MOG Antibody	Negative	Negative
IgG Index	0.48	0.00 - 0.77
CSF Culture	No growth after 3 days	No growth after 3 days
West Nile IgM Antibody	Positive	Negative
West Nile IgG Antibody	Negative	Negative
Eastern Equine Encephalitis (EEE) IgM Antibody	Negative	Negative
Meningitis/Encephalitis Panel by PCR *	Negative	Negative
Epstein Barr Virus PCR	Negative	Negative
Enterovirus by PCR	Negative	Negative
Encephalitis/Paraneoplastic Autoantibody Panel **	Negative	Negative
CSF Cytology	No malignant cells	No malignant cells
CSF Flow Cytometry	No abnormal lymphocyte population	No abnormal lymphocyte population

The patient received empiric intravenous immunoglobulin (IVIG) therapy at a dose of 400 mg/kg/day for five consecutive days (total dose of 2 g/kg), starting from the third day of his hospital stay. The patient also experienced moderate pain in the lower back with radiation to both legs on the fifth day of his hospital stay. He received gabapentin (300 mg orally three times daily) and responded favorably to the treatment. His clinical condition also improved markedly after finishing the IVIG course, as he no longer had a headache or double vision and could stand up and walk a few steps. He was discharged to an acute rehab facility where his symptoms continued to partially improve. Of note, the nerve conduction study (Table 3) and electromyography (Table 4) about four months after the onset of symptoms showed electrodiagnostic evidence of severe disorder of motor neurons and/or their axons in the thoracic and lumbosacral myotomes with signs of active denervating features.

**Table 3:** Nerve conduction studies (NCS) four months after the onset of symptoms in the presented case with West Nile virus infection. AHB, abductor hallucis; EDB, extensor digitorum brevis; NL, normal; NR, no response; TA, tibialis anterior.

Site	Latency (ms)		Amplitude (motor=mV; sensory=µV)		Conduction velocity (m/s)	
	Patient’s value	NL range	Patient’s value	NL range	Patient’s value	NL range
Right peroneal (EDB) motor						
Ankle	NR	<6.5	NR	>1.10	-	-
Below the fibular head	NR	-	NR	-	NR	>36
Lateral popliteal fossa	NR	-	NR	-	NR	≥42
Left peroneal (EDB) motor						
Ankle	4	<6.5	1.87	>1.10	-	-
Below the fibular head	13.7	-	1.65	-	36	>36
Lateral popliteal fossa	15.8	-	1.51	-	48	≥42
Right tibial (AHB) motor						
Ankle	NR	<6.1	NR	>5.3	-	-
Knee	NR	-	NR	-	NR	≥37
Left tibial (AHB) motor						
Ankle	6.9	<6.1	4.1	>5.3	-	-
Knee	17.1	-	3.5	-	41	≥37
Right peroneal (TA) motor						
Fibular head	NR	<4.2	NR	>2.9	-	-
Lateral popliteal fossa	NR	-	NR	-	NR	-
Left peroneal (TA) motor						
Fibular head	2.8	<4.2	4	>2.9	-	-
Lateral popliteal fossa	4.4	<5.7	3.6	-	63	-
Right sural sensory						
Calf-lateral malleolus	3.5	<4.5	8	>4	42	≥40
Left sural sensory						
Calf-lateral malleolus	3.3	<4.5	19	>4	46	≥40
Right superficial peroneal sensory						
Wrist-digit V	3.3	<4.2	6	>5	52	≥40
Left superficial peroneal sensory						
Forearm-wrist	2.8	<4.2	25	>5	70	≥50

**Table 4:** Electromyography (EMG) four months after the onset of symptoms in the presented case with West Nile virus infection. CRD, complex repetitive discharge; Fasc, fasciculation potentials; FDI, first dorsal interosseous; Fibs, fibrillation potentials; Incr, increased; Ins Act, insertional activity; MUAP, motor unit action potentials; NL, normal; Poly, polyphasic; PSW, positive sharp waves; Recrt, recruitment; Spon Disc, spontaneous discharges.

Muscle (All right side)	Ins Act	Fibs/ PSW	Fasc	Spon Disc	Amplitude	Duration	Poly	Recrt	Activation
Medial gastrocnemius	Incr	2+	NL	NL	-	-	-	No MUAP	NL
Lateral gastrocnemius	Incr	2+	1+	NL	-	-	-	No MUAP	NL
Tibialis anterior	Incr	1+	NL	NL	2+	2+	NL	Single MUAP	NL
Vastus lateralis	Incr	1+	NL	NL	2+	2+	NL	Single MUAP	NL
Vastus medialis	Incr	1+	1+	NL	2+	2+	NL	Single MUAP	NL
FDI	NL	NL	NL	NL	NL	NL	NL	NL	NL
Deltoid	NL	NL	NL	NL	NL	NL	NL	NL	NL
Mid lumbar paraspinal	Incr	1+	NL	CRD	-	-	-	-	-
Mid thoracic Paraspinal	Incr	2+	NL	NL	-	-	-	-	-

## Discussion

LETM is inflammation affecting the spinal cord and is specifically defined as lesions visualized on a spinal MRI with abnormal T2 signal traversing over three or more adjoining vertebral segments [9]. There are various known pathologies associated with LETM including infectious, granulomatous, neoplastic, and paraneoplastic diseases, acute disseminated encephalomyelitis, spinal cord infarction, dural arteriovenous fistula, and most commonly neuromyelitis optica (NMO) and NMO spectrum disorder [10]. Of the infectious causes, LETM has been outlined in the literature to be implicated in the para-infectious stage of various viral infections such as human immunodeficiency virus (HIV) [11] and human T-lymphotropic virus type I or II (HTLV-I/II) [12]. LETM could be a rare presentation of WNV infection [6,7]. On the other hand, leptomeningitis is a specific term referring to the inflammation of the two innermost layers of the meninges covering the brain and the spinal cord, the pia mater, and the arachnoid membrane [13,14]. Radiographically it is identified by contrast enhancement of the pia mater that extends into the subarachnoid spaces of the sulci and cisterns best visualized on post-contrast fluid-attenuated inversion recovery (FLAIR) images on MRI of the brain or spinal cord [15]. Leptomeningeal enhancement is usually associated with meningitis and the primary mechanism of the radiographic findings is due to the breakdown of the blood-brain barrier without angiogenesis [14]. In our presented case, given the positive WNV IgM antibody in both serum and CSF studies and negative other infectious, autoimmune, and inflammatory workup, the abnormal MRI findings are related to WNV neuroinvasion. Although the exact mechanisms underlying the WNV neuroinvasion are not completely understood, it is proposed that the WNV can enter the central nervous system (CNS) through two possible pathways: (i) the hematogenous route and (ii) the transneural route [4,16]. The hematogenous route involves the virus crossing the blood brain barrier (BBB), which can happen in two ways: (a) by direct infection of the endothelial cells that line the BBB and (b) by hitchhiking on WNV-infected leukocytes that migrate across a permeabilized BBB (this is known as the “Trojan Horse” mechanism) [4]. Transneural invasion of the CNS by WNV is suggested to occur either (i) from the peripheral somatic nerves into the CNS, or (ii) from the olfactory nerves into the CNS [4].

Clinically, our case outlines the ill-defined and non-specific nature of the presentation of West Nile neuroinvasive disease (WNND). WNND can pose a diagnostic challenge for the clinician and if unrecognized, is associated with significant morbidity and mortality in older and compromised individuals [17]. While a period of high fever is one of the most common clinical presentations of WNV infection, developing in approximately 25% of those infected, it can vary greatly in clinical severity. WNND, defined as the development of meningitis, encephalitis, or acute flaccid paralysis, develops in less than 1% but carries a fatality rate of approximately 10% [16]. Our patient also presented with non-specific systemic symptoms associated with WNV infection such as fever, headache, malaise, myalgia, and chills. Features of WNND that were present included symptoms of malaise, coarse tremors in his upper extremities, and difficulty walking that developed into progressive asymmetric paralysis. While CNS involvement is often found with these diseases, it is important to recognize that a minority of patients can also present without confusion or altered mental status, as outlined in this case report [14]. Ultimately, the diagnosis of WNND requires the presence of positive antibody titers in CSF [15].

WNV-associated paralysis most commonly results from the destruction of the anterior horn cells of the spinal cord, resulting in isolated temporary or permanent motor paralysis without a sensory deficit [13]. However, it has been noted that a minority of patients can have an atypical presentation with acute onset weakness or flaccid paralysis as a result of LETM. WNV LETM can uniquely present with asymmetric areflexic or hyporeflexic weakness with a preserved sensory response, consistent with anterior horn demyelination [5]. It is important to consider a broad differential diagnosis in such a presentation including Guillain-Barré syndrome (GBS) and poliomyelitis-like process, as differentiating between these pathologies clinically can be quite difficult. Primarily, the chronology of symptom development differentiates GBS from WNV or poliomyelitis as the symptom onset of GBS tends to occur weeks after an acute infection, whereas in WNV LETM occurs more frequently during the acute infectious period [5].

Fever and leukocytosis are prevalent manifestations in West Nile myelitis, in contrast to GBS, though in our patient leukocytosis was not present on initial evaluation. Furthermore, the pattern of paralysis can also aid in distinguishing the two conditions, as GBS typically presents with symmetrical paralysis [18], while West Nile myelitis manifests as asymmetrical paralysis. WNND can also often include encephalopathy, a characteristic absent in GBS, though, as outlined in this case report, there has been evidence of WNV myelitis presenting without encephalitis [5]. Electrodiagnostic studies can help objectively differentiate WNV myelitis and GBS as GBS is characterized by nerve conduction slowing or blocking due to demyelination whereas WNV myelitis shows significantly decreased or absent motor function from implication of the anterior horn cells [16,18,19]. Finally, CSF analysis indicates elevated protein levels in both conditions, but West Nile myelitis may exhibit pleocytosis, a distinction absent in GBS [18].

Data surrounding long-term neurologic and functional outcomes of patients infected with WNV is not well understood and is influenced by the severity of the presenting illness, preexisting comorbid conditions, and degree of involvement of neuroinvasive disease. Cognitive complaints in patients suffering from WNND such as difficulties with attention and concentration, have been well documented and suggest a subcortical origin of cognitive dysfunction based on prominent thalamic and basal ganglia involvement [19]. While the presence of preexisting comorbid conditions is associated with a longer recovery of physical and mental symptoms, the rates of recovery of participants with WNND compared to WNV non-neuroinvasive disease was similar in patients; and patients with WNND demonstrated at least a 95% recovery within a year after onset [14].

Unfortunately, there are no current antiviral treatment options for neuroinfection (e.g., encephalomyelitis) related to arboviruses, such as WNV, and the treatment involves mainly supportive care. Although there are some preclinical studies utilizing monoclonal antibodies neutralizing these viruses, there are no clinical studies in this regard. The utility of high-dose corticosteroids for the management of WNND is still controversial [20]. Evidence from some studies suggests that IVIG is beneficial for patients with WNV encephalitis, with a recommended dose of 0.4 g/kg for five days [20]. Our case also responded partially to IVIG therapy followed by discharge to an acute rehabilitation facility where he continued physical therapy.

## Conclusions

We described a rare case of WNV infection with concurrent LETM and leptomeningitis-related symptoms. This case highlights the importance of considering WNV infection in the differential diagnosis of patients who present with such manifestations. Serologic WNV antibody testing in both serum and CSF can help confirm the diagnosis if there is a clinical suspicion of WNV infection.

Patients with WNV infection and CNS involvement may benefit from IVIG therapy, which can help them recover faster from their symptoms. IVIG therapy should be started as soon as possible after diagnosing WNV-related LETM and leptomeningitis, which are serious complications of the infection. This is why prompt identification and treatment of these conditions are crucial.
